# A Case of Mesenteric Thrombosis

**Published:** 1906-09

**Authors:** G. Munro Smith

**Affiliations:** Surgeon to the Bristol Royal Infirmary


### A CASE OF MESENTERIC THROMBOSIS.

BY

G. Munro Smith, M.R.C.S.,

*Surgeon to the Bristol Royal Infirmary*

A. M., a healthy-looking man of forty-five, by trade a mason, was admitted to the Bristol Royal Infirmary on February 5th, 1906, complaining of abdominal pain and sickness. He said that he had always enjoyed good health and had never before had a similar illness. Sixteen days before admission, whilst at work, he was seized with pain in the belly, severe, but not bad enough to keep him from following his occupation, which he continued for two hours, after which he went home and to bed. He felt better the next day and got up to dinner. He ate some stuffed pork; this was followed by violent pains in the upper part of the abdomen and in the chest, and he vomited. He took to his bed and sent for a doctor, who gave him morphia, &c., and kept him on fluid diet. From this time onwards he vomited every day. The bowels were open four days before admission. When I first saw him, on February 6th, he was somewhat pale and anxious looking; the tongue was moist and furred at the edges; there was no distension; the abdomen was slightly tender on palpation; pulse soft, slow and regular; temperature 101.6° F. The next day the temperature was 97° F., pulse good, pain easier. He vomited some greenish mucus. An enema brought away a fairly large semi-solid motion, with no tinge of blood. Rectal examination revealed nothing.

On February 8th he was not quite so well, and on the 9th it was evident that he was losing ground, and it was decided to operate. On opening the abdomen, a coil of small intestine at once presented itself, rather distended, purplish in hue and mottled with blue and dull red patches. This condition extended over about three feet of the ileum. In the absence of any twist or band it was thought that this might be due to mesenteric thrombosis, especially as there was no sharp line of demarkation between the healthy and unhealthy gut; but no thrombosed vessels could be felt, neither the mesentery nor intestine were thickened or œdematous, and the distension was not marked. The question of resection of the affected intestine, or of an artificial anus was discussed; but there were signs of extension of the diseased condition to the neighbouring coils of intestine, and either operation seemed to me useless. His condition grew steadily worse, and he died the next day, three weeks from the initial symptoms.

At the *autopsy* it was found that the purple discolouration had extended to the whole of the small intestine, except the first and last portions. The mesentery was full of small thrombosed vessels. The superior mesenteric artery was occluded by a thrombus about four inches long extending from three to seven inches from the orifice of the vessel. The upper part of this thrombus was firm, the lower part was softer and more recent. The mesenteric veins were also extensively thrombosed. There was no cardiac lesion, and the other organs of the body were apparently healthy. No atheromatous patches could be found in the arteries.

About a hundred and seventy cases of mesenteric thrombosis have been recorded, the great majority being of the superior mesenteric vessels, not the inferior, the proportion being as great as forty to one. The arteries are more frequently blocked than the veins as five to one.

Why the mesenteric arteries should be especially liable to embolism or thrombosis is not clear; probably the fatal consequences of this lesion make the relative frequency compared with other arteries appear greater than it really is. And for the same reason thrombosis of the superior mesenteric vessels seems much more common than the same lesion in the inferior mesenteric, because in the former case the result is fatal; in the latter the symptoms may be comparatively trifling, and death seldom results

In this case no cause for the thrombosis was ascertained; the vessels were healthy, and microscopic examination of the artery at the seat of the clot revealed no degeneration of the arterial walls. There is, of course, always the possibility of syphilis, hereditary or acquired, to fall back upon; but there was no history of either in this instance.

There are no symptoms which enable a correct diagnosis to be made during life in such a case as this; and having made the diagnosis by opening the abdomen, there is little or nothing to be done for the patient's relief. Had excision of several feet of bowel been effected no good could have resulted, for a few hours after the exploratory incision was made, eight or nine more feet of small intestine were involved. Neither could an artificial anus have saved the patient, for it could not have averted the inevitable gangrene of nearly the whole of the ileum, nor the invasion of the necrosed tissue with the germs which infest the intestine; and it is probably as much the pyogenic organisms which work out the final result as the mere obstruction to the intestinal contents.

